# European trial of free light chain removal by extended haemodialysis in cast nephropathy (EuLITE): A randomised control trial

**DOI:** 10.1186/1745-6215-9-55

**Published:** 2008-09-28

**Authors:** Colin A Hutchison, Mark Cook, Nils Heyne, Katja Weisel, Lucinda Billingham, Arthur Bradwell, Paul Cockwell

**Affiliations:** 1 Department of Nephrology, University Hospital Birmingham, Birmingham, UK; 2 Division of Medical Sciences, University of Birmingham, Birmingham, UK; 3 Department of Haematology, University Hospital Birmingham, Birmingham, UK; 4 Department of Nephrology, University Hospital Tübingen, Germany; 5 Department of Haematology, University Hospital Tübingen, Germany; 6 Cancer Research UK Clinical Trials Unit, University of Birmingham, Birmingham, UK; 7 Division of Infection and Immunity, University of Birmingham, Birmingham, UK

## Abstract

**Background:**

Renal failure is a frequent complication of multiple myeloma and when severe is associated with a greatly increased morbidity and mortality. The principal cause of severe renal failure is cast nephropathy, a direct consequence of high concentrations of monoclonal free light chains (FLCs) in patients' sera. FLC removal by extended haemodialysis, using a high cut-off dialyser, has recently been described as a novel therapeutic option.

**Methods:**

The **EU**ropean trial of free **LI**ght chain removal by ex**TE**nded haemodialysis in cast nephropathy (EuLITE) trial is a prospective, randomised, multicentre, open label clinical trial to investigate the clinical benefits of FLC removal haemodialysis in patients with cast nephropathy, dialysis dependent acute renal failure and *de novo *multiple myeloma. Recruitment commenced in May 2008. In total, 90 patients will be recruited. Participants will be randomised, centrally, upon enrolment, to either trial chemotherapy and FLC removal haemodialysis or trial chemotherapy and standard high flux haemodialysis. Trial chemotherapy consists of bortezomib, doxorubicin and dexamethasone. FLC removal haemodialysis is undertaken with two Gambro HCO 1100 dialysers in series using an intensive treatment schedule. The primary outcome for the study is independence of dialysis at 3 months. Secondary outcomes are: duration of dialysis, reduction in serum FLC concentrations; myeloma response and survival.

**Hypothesis:**

FLC removal haemodialysis will increase the rate of renal recovery in patients with severe renal failure secondary to cast nephropathy in *de novo *multiple myeloma.

**Trial registration:**

ISRCTN45967602

## Background

Renal impairment is a frequent complication of multiple myeloma affecting between 18 and 56% of patients at presentation [1-4]. When severe it is associated with a greatly increased morbidity and mortality. However, improvement of renal function also improves the patients' survival [2,5,6]. Previous studies have demonstrated that the principal cause of severe renal failure in patients with multiple myeloma is cast nephropathy, a direct consequence of the high concentrations of monoclonal free light chains (FLCs) in the patients' sera.

Treatment strategies for patients with severe renal failure and multiple myeloma should first focus on correcting reversible factors such as dehydration and hypercalcaemia [7]. Second, disease specific treatments should be initiated rapidly and there is growing evidence for the role of new chemotherapeutic agents [8-10]. The third treatment option for these patients is direct removal of monoclonal FLCs from the serum. Historically, this has been undertaken using plasma exchange, however the efficacy of this treatment has never been established [11-13].

Recently we described the removal of FLCs by extended haemodialysis, using the Gambro HCO 1100, as an alternative strategy [14]. Results of early pilot studies suggest that the procedure is not only effective at removing FLCs, but it is also associated with improved clinical outcome [6,15]. In 17 patients with cast nephropathy treated, 12 recovered sufficient renal function to become independent of dialysis. This rate was a significantly higher than that of a case matched control population from the same institution, P < 0.01 [6].

The purpose of the EuLITE trial is to evaluate, in a controlled setting, FLC removal haemodialysis in patients with cast nephropathy, dialysis dependent renal failure and *de novo *multiple myeloma.

## Methods/design

### Hypothesis

The EuLITE trial examines the hypothesis that FLC removal haemodialysis will increase the rate of renal recovery in patients with cast nephropathy, severe renal failure and *de novo *multiple myeloma.

### Design

**Figure 1:**
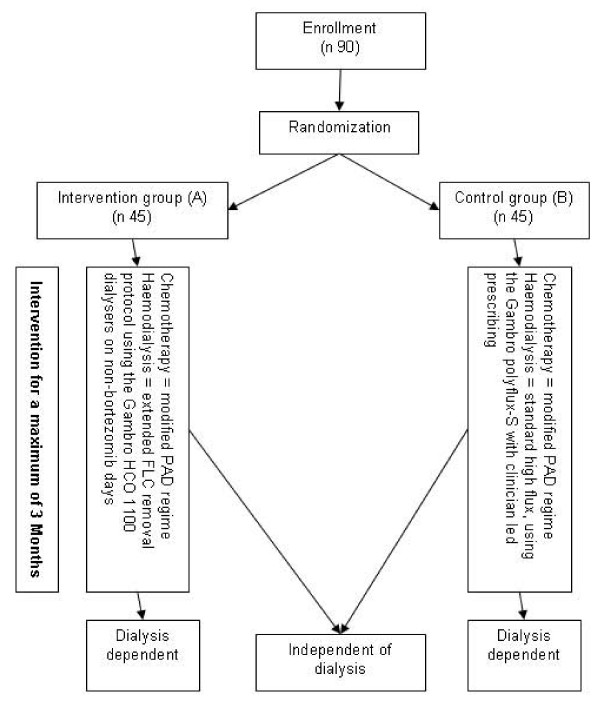
Trial Schema.

The EuLITE trial is a prospective, randomised, multicentre, open label clinical trial to investigate the clinical benefit of FLC removal haemodialysis in patients with cast nephropathy, dialysis dependent renal failure and *de novo *multiple myeloma [16]. Inclusion and exclusion criteria are detailed in Table 1. At enrolment patients are randomised into one of two treatment groups (Figure 1):

**Table 1:** Inclusion and exclusion criteria

**Inclusion criteria**
∘ Age greater than 18 years
∘ Dialysis dependent acute renal failure (defined by: an estimated GFR of < 15 ml/min/1.73 m^2^)
∘ Fulfils criteria for the diagnosis of symptomatic *de novo *multiple myeloma
∘ Abnormal serum FLC ratio and a serum FLC concentration > 500 mg/L
∘ Myeloma kidney demonstrated on a renal biopsy (cast nephropathy)
∘ Commencement of study within 10 days of presenting to enrolling unit

**Exclusion criteria**

∘ Known advanced chronic renal failure (eGFR < 30 mls/min/1.73 m^2^) or evidence of significant chronic damage on renal biopsy
∘ Amyloidosis or light chain deposition disease on renal biopsy
∘ Previous treatment of multiple myeloma with chemotherapy
∘ Haemodynamic instability that precludes unsupported renal replacement therapy
∘ Significant cardiac disease
∘ Advanced disease or significant co-morbidity: with poor short term prognosis, necessitating palliation and no active or disease specific treatment.
∘ History of allergic reaction attributable to compounds containing boron or mannitol
∘ Peripheral neuropathy or neuropathic pain Grade 2 or higher
∘ Clinically significant liver dysfunction (bilirubin > 1.8 mg/dl (30 umol/L))
∘ Active uncontrolled infection or known HIV infection
∘ Inability to give informed consent
∘ Pregnant and lactating women

Group A (intervention): This group will receive extended haemodialysis using Gambro HCO 1100 dialysers and standard trial chemotherapy of: bortezomib, doxorubicin and dexamethasone.

Group B (control): This group will receive standard high flux haemodialysis (using a Gambro Polyflux-S dialyser) at intervals determined on clinical grounds by the nephrologist supervising care (minimum 4 hours 3 times per week) and standard trial chemotherapy of: bortezomib, doxorubicin and dexamethasone.

Randomisation is undertaken using random number generation by the programme Datinf RandGen (Datinf, Tubingen, Germany). The minimization algorithm described by Pockock and Simon [17] was employed to ensure balance between the intervention age groups (18–55, 56–65, 66–75 and 75+) and study centre. The allocation is made at random with a weighting in favour of the intervention that would minimize imbalance with a probability of 0.8. The seed for random number generation is stored securely to allow reproduction of the random numbers if required.

### Study chemotherapy (both groups)

The first six patients entered into the study will comprise a cohort assessed for the safety of a reduced dose PAD regimen [18]. The modified regimen will consist of: bortezomib (1 mg/m^2 ^on days 1, 4, 8 and 11 of a 21 day cycle); doxorubicin (9 mg/m^2 ^on days 1 and 4 of the 21 day cycle) and dexamethasone (40 mg daily days 1–4, 8–11 and 15–18 for the first 21 day cycle and days 1–4 only on subsequent cycles). This safety assessment will be independent of the renal therapy to which they have been assigned.

Supportive therapy:

∘ Co-trimoxazole 480 mg three times per week or alternative *Pneumocystic carinii *prophylaxis as per local institutional practice.

∘ Ranitidine 150 mg twice daily whilst on dexamethasone

∘ Metoclopramide 10–20 mg three times per day as required (20 mg to be given pre-chemo on day 1)

∘ Allopurinol 100 mg orally daily

∘ Aciclovir 200 mg twice per day, orally

Chemotherapy may have to be stopped or withheld for medical reasons. Such patients will be withdrawn from the treatment group and revert to standard care for their dialysis unit. Patients who withdraw for any reason will still be followed-up for all outcome measures, in order to enable an 'intention to treat analysis'.

### FLC removal haemodialysis (group A only)

Participants will receive haemodialysis on non-bortezomib days: 6 hours on day 0, then 8 hours on days 2, 3, 5–7, 9+10. After day 12, participants will receive 8 hours of haemodialysis on alternate days. Dialysis will be undertaken using two Gambro HCO 1100 dialysers in series at a blood flow of 250 ml/min, dialysate flow of 500 ml/min, with adequate heparin (activated clotting time of 180–200 seconds). The supervising clinician will determine the fluid removal, with the aim to not dehydrate the patient.

Extended FLC removal haemodialysis will be supported by replacement of human albumin solution, in the last hour of each dialysis session (60 g). Magnesium, calcium and potassium will be replaced as required. After day 21, if patients still requires renal support the dialysis schedule will be reduced to 6 hours three times per week.

### Primary endpoint

The primary endpoint of the study is independence of dialysis at three months from enrolment. Independence of dialysis will be defined as an estimated GFR of > 15 mls/min/1.73 m^2^, 2 weeks after the last dialysis session.

Patients' renal function will be assessed by biochemical markers (urea, creatinine and an estimated GFR) with monitoring of urine output. Patients who have a pre-dialysis eGFR of > 20 ml/min and a good urine out-put should receive a trial with out dialysis. The nephrologist responsible for the patients' care will decide when this is suitable. Patients who become independent of dialysis during the first 90 days, but subsequently require further dialysis support, so that they are dialysis dependent at the 3 month point will be classified as treatment failures.

### Secondary endpoints

The first secondary endpoint is to determine the efficiency of FLC removal HD that results in sustained reductions in serum FLC concentrations versus a standard dialysis. Serum FLC concentrations will be measured pre- and post-dialysis, daily for the first 21 days on dialysis days.

It is proposed that patients with a cast nephropathy will recover their renal function more rapidly if their serum FLC concentrations are reduced quickly. We will, therefore, observe the duration of requirement for HD.

The management of patients with multiple myeloma is limited by renal impairment. It is conceivable that patients who recover renal function will have an increased chance of being suitable for high dose melphalan and stem cell transplantation, which is the current standard of care after primary chemotherapy. Response to treatment of multiple myeloma will be documented at the end of each chemotherapy cycle, then 6 and 12 months. Whether patients under 70 years of age have received a stem cell transplant will be recorded at 12 months.

Renal failure is the second commonest cause of death in patients with multiple myeloma. Therefore, patients who recover renal function should have a lower mortality rate. Overall survival will be recorded in both arms of the study.

### Case report forms and data collection

All study data will be recorded on electronic Case Report Forms (eCRF) which are generated on a dedicated randomisation and remote data entry web server (Datinf, Tubingen, GmbH). The CRFs will be completed and signed by the local investigator or his/her designee as soon as the requested information is available. During the first 21 days, eCRFs will be completed for each dialysis day. These document the details of dialysis (including any breaches in protocol), biochemistry and haematological data. A specific eCRF is completed at the end of each chemotherapy cycle to document compliance with chemotherapy protocol. Time points for clinical and laboratory data are at the end of each cycle of chemotherapy and 3, 6, and 12 months.

#### Chemotherapy response

After a maximum of 4 cycles of treatment, patients will be assessed for response to PAD chemotherapy according to the International uniform response criteria for multiple myeloma [19]. If there is no evidence of response i.e. stable disease or progressive disease, then the patient will have been deemed to have failed PAD chemotherapy, and treatment will be switched to a regimen compatible with local institutional practice. Patients with a partial response or very good partial response will be eligible to continue for a maximum of 8 cycles. Patients who have achieved a stringent complete response or complete response will be eligible to stop chemotherapy and proceed to autologous peripheral blood stem cell collection if considered suitable for high dose therapy and stem cell rescue.

### Statistical analysis plan

The primary analysis for the trial is to compare the experimental treatment with the control treatment in terms of independence of dialysis at three months. The proportion of patients independent of dialysis will be compared using point and interval estimates of relative risks and continuity-adjusted chi-square tests. The analysis will be carried out on an intention-to-treat basis. Due to the short follow-up required for the primary endpoint, missing data are not expected but the analysis will include these as failures and sensitivity analysis will assess the robustness of the conclusions to this assumption. As an additional analysis, time taken for independence of dialysis to occur will be compared between the two groups. Times will be censored for those patients who are not independent of dialysis at the time of analysis or lost to follow-up. Kaplan-Meier estimation and log-rank tests will be used to compare the groups.

The change in serum FLC concentrations over time will be compared across the two groups using longitudinal analysis with imputation used for any missing data. In particular, the treatment groups will be compared in terms of changes in mean pre-dialysis serum FLC concentrations for days 4–6, 8–10 and 20–22 relative to the pre-randomization level using analysis of variance. The appropriate parametric or non-parametric version of this analysis will be determined by graphical assessment of the normality of the data. Myeloma response at 6 and 12 months, and the proportion to have undergone a peripheral blood stem cell transplant at 12 months, will be compared across treatment groups using estimates of relative risks and continuity-adjusted chi-square tests. Mortality will be documented for a follow-up period of 24 months. Survival time will be censored for those patients still alive at the time of analysis or lost to follow-up and Kaplan-Meier estimation and log-rank tests will be used to compare treatment groups.

The balance of baseline factors across the two treatment groups will be assessed descriptively as hypothesis testing is not appropriate in this randomised setting. The key baseline factor in this setting is age which will be stratified for in the randomisation procedure. Analysis of the primary outcome measure will adjust for age using logistic regression modeling to ensure that the validity of the conclusions. Appropriate modeling will also be used to adjust for baseline factors in the analysis of any secondary outcomes.

#### Timing of analysis

Interim analysis will be carried out 3 months after the first 25 patients have been recruited and then when recruitment of 50% of target has been achieved. Data will be presented to an independent Data Monitoring Committee. The interim analysis will present recruitment data and data on outcomes and adverse events. Given the small size of the study, the interim analysis will be purely descriptive with the primary aim to assess patient safety and that it is ethical to continue. The study is expected to complete recruitment within 1 year. Final analysis will be carried out after all patients have been followed up for a minimum of 3 months and a further analysis of the survival outcome will be carried out after all patients have been followed-up for a minimum of 24 months.

#### Calculation of sample size

Sample size calculations are based on the primary outcome of independence of dialysis after 3 months from enrolment. Data from previous studies suggest a recovery rate of 25% in the control group and suggest that an improvement to 55% in the treatment group is a realistic expectation. Assuming an alpha error level of 5%, 41 patients are required in each treatment arm to detect this size of difference with 80% power. The primary analysis will be on an intention-to-treat basis but to enable sufficient power for any per-protocol analysis the final target is increased by 10% to allow for patients who withdraw from protocol treatment at the discretion of the treating physician. These patients will be followed-up for all outcome measures unless consent is withdrawn. The target recruitment is therefore 45 patients per arm, 90 patients in total.

### Safety assessments

Safety assessments will include the monitoring and recording of all adverse events, including serious adverse events. Serious adverse events that are unexpected and considered by the investigator to be related to bortezomib will be reported to the ethics committee and the Medicines and Healthcare Products Regulatory Agency. Expected adverse events are presented in Table 2, unexpected are those not given. Grading and causality of the events will be undertaken by the site investigator and reviewed by the principal investigator.

**Table 2:** Expected adverse events

**Dialysis related**	**Chemotherapy related**
∘ Hypotension during dialysis	∘ Infections and infestations
∘ Dialysis line exit site infection	∘ Neoplasms benign, malignant and unspecified
∘ Cardiac arrhythmia	∘ Blood and lymphatic system disorders
∘ Septicaemia	∘ Immune system disorders
∘ Endocarditis	∘ Endocrine disorders
∘ Fluid overload	∘ Metabolism and nutrition disorders
∘ Peripheral oedema	∘ Psychiatric disorders
∘ Hypoalbuminemia	∘ Nervous system disorders
∘ Hypophosphatemia, hypomagnesemia, hypokalaemia, hypocalcaemia	∘ Eye disorders
∘ Hypersensitivity reactions	∘ Ear and labyrinth disorders
	∘ Cardiac disorders (including cardiac tamponade)
	∘ Vascular disorders
	∘ Respiratory, thoracic and mediastinal disorders (including Pneumonitis, interstitial pneumonia, Acute Respiratory Distress Syndrome)
	∘ Gastrointestinal disorders (including ischemic colitis and liver failure, hepatobiliary disorders)
	∘ Skin and subcutaneous tissue disorders
	∘ Musculoskeletal and connective tissue disorders
	∘ Renal and urinary disorders
	∘ Reproductive system and breast disorders
	∘ Herpes meningoencephalitis
	∘ Angioedema
	∘ Encephalopathy, autonomic neuropathy
	∘ Ophthalmic herpes

## Summary

This trial will investigate the potential role of FLC removal haemodialysis in the management of patients with new multiple myeloma, cast nephropathy and dialysis dependent renal failure. If efficacious this therapy will offer clinicians new options in the management of these patients with historically very poor outcome.

## Competing interests

The sponsor for this study is the University Hospital Birmingham, Birmingham, U.K. The study is partially funded by Gambro, Hechingen, Germany who would be interested in a positive study result. The drug, bortezomib, is being provided free of charge for the study participants by Ortho-Biotech, High Wycombe, UK. The Binding Site, Birmingham, UK is providing the immunoassays to measure FLCs free of charge for this study. ARB is a Director of The Binding Site. LB will receive an education grant from Gambro for the time contributed to the study. The remaining authors do not receive any reimbursement or financial benefits from these organisations.

## Authors' contributions

CAH, MC, NH, KW, LB, ARB and PC all contributed to the study design, drafting of the protocol and this manuscript. In particular: LB is the trial statistician; MC and KW have developed the haematological care pathways for patients within the trial; CAH, NH and PC have developed the FLC removal by HCO-HD protocol. ARB has provided advice regarding measurement of FLCs within the study. All authors have read and approved the final manuscript.
